# Patients’ and family members’ experiences with cascade testing for Lynch syndrome in the USA: a qualitative interview study

**DOI:** 10.1007/s12687-026-00878-8

**Published:** 2026-03-23

**Authors:** Natalie P. Stewart, Eliza K. Courtney, Megan C. Roberts, Erin Turbitt

**Affiliations:** 1 Graduate School of Health, University of Technology, Sydney, NSW Australia; 2 Children’s Cancer Institute, Lowy Cancer Centre, UNSW Sydney, Kensington, NSW Australia; 3 Kids Cancer Centre, Sydney Children’s Hospital, High Street, Randwick, Sydney, NSW Australia; 4 School of Clinical Medicine, UNSW Medicine and Health, UNSW, Kensington, NSW Australia; 5 University of North Carolina Eshelman School of Pharmacy, Chapel Hill, NC USA; 6 Garvan Institute of Medical Research, Sydney, NSW Australia

**Keywords:** Cascade testing, Lynch syndrome, Hereditary cancer, Patient experience, Family experience, Genetic counseling

## Abstract

Cascade testing for Lynch syndrome is critical for the identification of at-risk relatives who may benefit from early detection and risk-reduction strategies. Uptake of cascade testing within families has consistently remained low, and strategies developed to address this have had varying degrees of success. Limited research exists that investigates the perspectives and lived experiences of individuals with or at risk of Lynch syndrome, particularly through rich qualitative methods in the context of cascade testing. This study aimed to explore the lived experience of patients and relatives with cascade testing for Lynch syndrome in the USA. We analyzed qualitative interviews performed with twenty patients either diagnosed with Lynch syndrome, or with a family member diagnosed with Lynch syndrome, using reflexive thematic analysis. Three overarching themes were developed: (1) logistics of disclosure, 2) emotions and beliefs surrounding cascade testing, and 3) reflections on strengths and limitations cascade testing. Genetic counseling was an important component of the cascade testing process; however, participants often described feeling under supported. We propose that alternative models of service provision could help to address this lack of support, and therefore assist in optimizing uptake of cascade testing for Lynch syndrome.

## Introduction

Lynch syndrome is a cancer predisposition syndrome caused by germline heterozygous pathogenic variants in mismatch repair (MMR) genes including *MLH1*, *MSH2* (including *EPCAM* deletions), *MSH6*, and *PMS2* (Lynch et al. 2015). Lynch syndrome is characterized by an increased lifetime risk for predominantly colorectal and endometrial cancers, as well as gastric, ovarian, urothelial, small bowel, and prostate cancers. Penetrance estimates vary considerably depending on the MMR gene involved (Dominguez-Valentin et al. 2020; Møller et al. 2018). Well established risk management guidelines exist for Lynch syndrome, involving surveillance and risk-reducing strategies. Regular colonoscopies were once thought to dramatically reduce the incidence of colorectal cancer among Lynch syndrome patients; however, research suggests variable impact on incidence depending on the type of MMR mutation carried, as well as quality and frequency of colonoscopy (Dominguez-Valentin et al. 2020; Helderman et al. 2024; Møller et al. 2022; Sánchez et al. 2022). Regular colonoscopies still greatly reduce mortality from colorectal cancer compared with no surveillance (Helderman et al. 2024; Seppälä et al. 2021). Daily use of aspirin significantly reduces the risk of colorectal cancer among individuals with Lynch syndrome (Burn et al. 2020). There are currently no effective early detection strategies for Lynch-related gynecological cancers. As such, risk-reducing hysterectomy and bilateral salpingo-oophorectomy are recommended after age 40 to 50 years (depending on the gene) or post-childbearing (Lim et al. 2022; Seppälä et al. 2021). Early identification of Lynch syndrome enables timely implementation of surveillance and risk-reducing strategies where available which aids in reducing cancer-related morbidity and mortality (Møller et al. 2018, 2022; Seppälä et al. 2021).

Globally, guidelines recommend routine screening of colorectal and endometrial tumor tissues for biological markers of Lynch syndrome, such as immunohistochemistry for MMR proteins, followed by diagnostic germline genetic testing (Giardiello et al. 2014; Kang et al. 2020; Seppälä et al. 2021). Estimates suggest that up to 98% of individuals with Lynch syndrome may be unaware of their condition and likely have reduced access to surveillance and risk-reducing measures (Actkins et al. 2021; Hampel and Chapelle 2011). These estimates are based on the prevalence of Lynch syndrome among colorectal cancer diagnoses, penetrance of MMR pathogenic variants, and low uptake of germline testing.

We define the cascade testing process as the one that starts with providing genetic risk information to relatives (disclosure), through to relatives engaging with a genetics service (engagement), and undergoing genetic testing (uptake). The cascade testing process enables appropriate risk management for individuals identified to carry a pathogenic MMR variant, and can exclude Lynch syndrome in those found not to carry the variant (Baroutsou et al. 2021).

Uptake of cascade testing demonstrates cost-effectiveness globally including in the USA, Australia, and Singapore (Breheny et al. 2006; Mvundura et al. 2010; Wang et al. 2012). Guidelines from the USA recommend testing for first- and second-degree relatives of a proband with Lynch syndrome; however, estimates suggest only 52% of first-degree relatives undergo cascade testing (Mvundura et al. 2010; Practice and Group 2009). A study in Singapore found only 12% of first-degree relatives underwent cascade testing for clinically actionable conditions including Lynch syndrome, which increased to just 20% when cascade testing was offered free of charge (Courtney et al. 2019). A more recent systematic review found that only around one third of relatives undergo cascade genetic testing when patients were responsible for disclosure (Frey et al. 2022). Multilevel barriers to uptake of cascade testing have been previously described and include low awareness about the condition, lack of provider follow-up, and fear of insurance and employment discrimination (Srinivasan et al. 2020a). Beyond systemic barriers, uptake of cascade testing may also be shaped by the rapid advancement of genomic technologies which increasingly frame genetic information as a prerequisite for autonomous health management. This shift fosters a familial ‘moral imperative’ in which pursuing testing is seen as the only responsible choice. Family members who decline testing may be marginalized, as their decision is seen as a failure of duty rather than a valid exercise of autonomy (Cowley 2016). Growing understanding of these multi-level barriers has prompted research focused on interventions to improve access, for example the ‘Let’s Talk’ intervention, an educational booklet provided to families to support understanding, communication, and decision-making (Passero et al. 2022).

Previous research has largely sought to explore patient preferences and patterns of family communication about genetic risk information using quantitative methodology. There is a lack of research involving rich qualitative exploration of the lived experience of individuals, specifically with or at-risk of Lynch syndrome, with cascade testing. Furthermore, little is known about the uptake of cascade genetic testing for hereditary cancer syndromes among racial and ethnic minorities, and there is a pressing need to conduct research focusing on diverse populations in this context (Ahsan et al. 2023). The insights gained from such research would be beneficial in informing the continued development of interventions to address identified barriers and leverage facilitators to increase access to cascade testing and appropriate risk management. Therefore, in our study we aimed to explore the experiences of patients, and their family members, with cascade testing for Lynch syndrome.

## Methods

### Theoretical and research approach

This qualitative study used a constructivist lens to appropriately address the aim of critically exploring lived experience from the perspectives of individuals (Schwandt 1994).

### Design

We performed qualitative analysis of interviews (*n* = 20) with Lynch syndrome patients or their relatives that were collected as part of a larger separate study investigating barriers and facilitators for cascade testing (Srinivasan et al. 2020a, b).

### Study participants

Of 60 interviews with key stakeholders part of the larger study, 20 were with people with Lynch syndrome or with a first-degree relative with Lynch syndrome. The research team used purposive sampling in the larger study to recruit a sample demographically diverse for gender, ethnicity, and race. Patients with Lynch syndrome and their relatives were recruited through an advertisement on a social media page for the patient advocacy and education organization Lynch Syndrome International (LSI). Eligible participants were at least 18 years old, had a diagnosis of Lynch syndrome or a relative with Lynch syndrome, lived in the USA, spoke English, and responded to a research study advertisement through the LSI social media page. There were no exclusion criteria. Eight participants were adults who were the first to undergo genetic testing for Lynch syndrome in their families (termed probands). Seven of these probands received a positive genetic test result and one tested negative. Twelve participants were adult blood-relatives of someone who had received a genetic diagnosis of Lynch syndrome (termed family members). Eleven of these blood-relatives had undergone cascade testing; ten had received a positive genetic test result for Lynch syndrome, and one had tested negative.

### Study measures

Researchers conducting the larger study developed a semi-structured interview guide to investigate factors that influence the implementation of cascade testing in clinical practice, informed by the Consolidated Framework for Implementation Research (CFIR) and the Integrated Behavioral Model (IBM) (Damschroder et al. 2009; Montano and Kasprzyk 2015). Using the CFIR enabled a comprehensive exploration of barriers and facilitators to the implementation of cascade testing across individual, intervention-related, organizational, and sociopolitical domains, while the IBM aided in targeting the guide to investigate individuals’ willingness to engage with cascade testing. Stakeholders (patient advocates and clinical experts) provided input to assist with the development of the interview guide which has been previously published elsewhere (Srinivasan et al. 2020a, b). Interview questions explored characteristics of the individual, experiences with cascade testing, and barriers and facilitators for cascade testing.

### Data collection and analysis

Interviews were conducted by telephone. Each interview was completed in one session, and all were digitally audio recorded between December 2018 and June 2019. All interviews were professionally transcribed and de-identified. On average, interviews lasted 60 min. All participants received a gift card on completion of the interviews as compensation for their time.

We performed data analysis using an inductive reflexive thematic analysis approach, because this is suited to investigate individuals’ experiences, perspectives, and for identification of behavioral patterns (Braun and Clarke 2022). We acknowledged researcher subjectivity as a primary tool in this study, regonizing inherent subjectivity in our qualitative data analysis and highlight that themes have been developed through coding by the researcher.

The reflexive analytical process was non-linear and recursive (Clarke et al. 2016). Two researchers (NS and ET) independently coded two interview transcripts and discussed differences in coding. Following this discussion, one researcher (NS) coded the remaining transcripts. While the process of co-coding is not necessarily consistent with the reflexive thematic analysis approach, we performed this step to upskill the student researcher (NS) in this process of analysis. Reflexive thematic analysis of the data set was supported by regular discussion and cross-examination with the broader study team, which provided a range of perspectives on the analysis and deepened the theme development.

## Results

Of the twenty individuals who participated, 60% (*n* = 12) were female, the mean age was 44.4 years (range 23–68), 60% (*n* = 12) identified as White/non-Hispanic/Spanish/Latino, 20% (*n* = 4) identified as White/Hispanic/Spanish/Latino, 5% (*n* = 1) identified as Black/African American, 5% (*n* = 1) identified as Black/African American/White, 5% (*n* = 1) identified as American Indian/Alaska Native/White, and 5% (*n* = 1) identified as American Indian/Alaska Native/Hispanic/Spanish/Latino. 85% (*n* = 17) were molecularly confirmed to have Lynch syndrome.

We developed three themes from the data: (1) The logistics of disclosure, (2) Emotions and beliefs surrounding cascade testing, and (3) Reflections on the strengths and limitations of current approaches to cascade testing. Exemplar quotes have been provided.

### Theme 1: logistics of disclosure

Participants reported variable experiences with disclosure of genetic risk information to their relatives. Participants described different approaches to communication and information dissemination depending on their perceived needs and those of other family members. They also discussed the availability, or lack, of written support materials about Lynch syndrome and cascade testing and the importance of these materials in aiding the process of disclosure.

#### Sub-theme 1a: modes and networks of communication

Participants described different modes of communication with family members. Some communicated with family by email, social media facilitated messaging, or text message, while others engaged by phone call, or in person.

*“I talked to my immediate family on the phone a lot and sent this comprehensive email and then*,* talked to some more extended family members*,* and*,* you know*,* I’ve reached everyone that I am aware of that is living … So*,* I called and spoke to my father’s sibling who was positive*,* and I emailed the children - my cousins.” -* Participant 154, female, non-Hispanic/Spanish/Latino, Lynch syndrome positive, first tested in family.

Other participants noted that social media, such as Facebook, was a useful tool for communicating with more distant family members with whom they would not regularly speak.

*“Facebook made it a little bit easier. It was easier to reach out and just be able to message my aunt than it would have been to call her.”* - Participant 479, non-Hispanic/Spanish/Latino, female, Lynch syndrome positive, cascade tested.

One participant noted their cultural customs meant that the acceptable mode of communication for receiving medical information was in-person.

*“With my family in particular*,* we are of Latin descent*,* so we don’t do well receiving that kind of information in texts or writing. It really has to be kind of in person*”- Participant 621, non-Hispanic/Spanish/Latino, female, Lynch syndrome positive, cascade tested.

The process of communicating with relevant family members was not always direct but involved networks of communication. Participants described being guided by family relationship dynamics when deciding with whom to disclose information. One participant described the utility of speaking with a non-blood relative, for the appropriate information to reach at-risk relatives effectively and encourage testing in those family members with whom communication was difficult.

*“I also let my stepmother know as well because my brother and sister weren’t too keen on having any tests done*,* and they kind of pooh-poohed [sic.] it and all of this and not - that was another issue. My oldest brother ended up going and getting screened after I got my positive*,* and he is also positive.”* – Participant 920, non-Hispanic/Spanish/Latino, female, Lynch syndrome positive, cascade tested.

#### Sub-theme 1b: the importance of support

Some participants explained that better support and more detailed guidance from their healthcare professionals would have helped them to better promote cascade testing amongst their relatives. Participants explained that verbal information was difficult to understand and retain, and that written information would have simplified and supported the process of cascade testing.

*“… A little guide that says how to talk to your family about Lynch Syndrome and testing and also some more information about Lynch Syndrome ‘cause it was kind of like a quick*,* crash course once my testing came back…I was in shock and so*,* didn’t hear everything that she said*,* and it would have been helpful to have*,* you know*,* a pamphlet or something saying*,* you know*,* these are the cancers that are linked to Lynch Syndrome*,* and this is the testing schedule most everyone should follow.”* – Participant 497, non-Hispanic/Spanish/Latino, female, Lynch syndrome positive, cascade tested.

Another participant highlighted that it would have been useful had they received information about how to approach family members in different age groups, with information that was appropriate to their stage of life:

*“I think that the primary one that I would say and would love to see myself is kind of giving people the tools and how to have that conversation with family members of different age groups… How to tell someone who’s in their 20s*,* 30s*,* 40s and elders. I think that if I would have known a better way of how to explain this to them*,* it would have really encouraged me to go a little bit further in my conversations.”* – Participant 621, non-Hispanic/Spanish/Latino, female, Lynch syndrome positive, cascade tested.

Many participants discussed how written support materials were used in their process of cascade testing. Several participants expressed they were not given adequate support or direction by their healthcare professionals entailing how to approach cascade testing. Some recalled they did not receive a notification letter to distribute to family members. As such, the information they shared reflected their personal understanding gained from their genetic counseling appointment, which the following participant felt was inadequate:

*“I don’t remember getting anything that you could take and hand to somebody and walk through and say this is what we got goin’ on*,* you know. Nothin’ I could share with*,* like*,* say*,* my daughter. You know*,* she just knows the best way I know how to explain it.”* – Participant 459, Hispanic/Spanish/Latino, female, Lynch syndrome positive, first tested in family.

Participants expressed a desire for a document to pass on to other family members outlining the information pertinent for cascade testing in an accessible fashion, referred to as a family notification letter.

*“I think that might have been helpful to actually have a letter and maybe a little trifold brochure that can fit in an envelope together that you can mail out just so that they’ve got the information of*,* like*,* okay*,* this is what it increase your risks for; because you have a family member that’s already been diagnosed*,* you have a 50/50% chance of having it and then*,* like*,* maybe websites or phone number for*,* like*,* the genetic counselors.”* - Participant 142, non-Hispanic/Spanish/Latino, female, Lynch syndrome positive, first tested in family.

Some participants explained that they received encouragement from trusted friends with a medical background. This informal, perceived expert support was an important part of the cascade testing process. One participant described reaching out to a friend from high school, a geneticist, and the impact of this friend’s support on her approach to cascade testing.

*“It was very helpful. That was what really got my ball rolling*,* and I felt much more informed about the cascade testing and better ways to handle it.”* – Participant 644, non-Hispanic/Spanish/Latino, female, Lynch syndrome positive, first tested in family.

### Theme 2: emotions and beliefs surrounding cascade testing

Participants expressed emotion in their descriptions of their cascade testing experiences. Participants often recounted a strong desire for all at-risk relatives to undergo genetic testing, frustration in the face of resistance from family members, and suggested an underlying strong belief that cascade testing may be lifesaving. Participants described their irritation towards, and disapproval of, those family members who did not pursue predictive genetic testing. The following participant strongly condemned any desire ‘not to know’ among relatives.

*“I hate this attitude - his son said ‘well*,* I don’t wanna know. I just don’t wanna know if I got it or not.’ Well*,* that’s terrible. That’s a terrible attitude. …”* – Participant 203, non-Hispanic/Spanish/Latino, male, Lynch syndrome positive, first tested in family.

A sense of resignation was evident among some participants when describing their efforts with cascade testing. Participants explained that once they had shared relevant information with family members, they had played their part in cascade testing and felt they had done all they could to promote cascade testing uptake. The following participant felt that cascade testing was important, but her responsibility ended at the point of disclosure:

*“She said she would mention it to her doctor*,* but I don’t know if she followed-up and did that. I*,* finally*,* you know*,* quit worrying about everybody. I’ve told everybody. ‘I did what I could.’”* – Participant 467 female, non-Hispanic/Spanish/Latino, Lynch syndrome positive, first tested in family.

One participant explained that after some encouragement from her cancer geneticist, she felt motivated to re-contact her father who had previously not engaged with genetics services.

*“I went to the genetic oncologist here at the local hospital*,* she encouraged me to ask him again*,* and I did*,* and I felt like I had more information that she had provided*,* and said*,* you know*,* this is what she said. You know*,* they’re not saying you have to change your health care routine or anything that you wanna do. It’s completely up to you*,* but it would just help us with our children and our family to make sure there’s nothing else going on.”* – Participant 644, non-Hispanic/Spanish/Latino, female, Lynch syndrome positive, first tested in family.

Many participants spoke about cascade testing for Lynch syndrome as potentially or actually lifesaving. For the following participant, the sense that cascade testing could have been lifesaving for his late brother, who died of a Lynch-related cancer, meant he felt determined to promote this lifesaving effect by endorsing uptake of cascade testing among his remaining relatives:

*“My intention is to save lives here because I lost my brother to colon cancer*,* and he was younger to me - I mean*,* younger than me. And*,* there’s a lot of people that in my family that are not going to the doctor*,* and I just like*,* you know*,* driving the point home that we can do this.”* – Participant 902, Hispanic/Spanish/Latino, male, Lynch syndrome carrier, cascade tested.

Another participant described how he believed his insistence for his niece to engage with genetics services and undergo genetic testing saved her life. Her mother, the participant’s sister, died of Lynch-related cancer. Undergoing cascade testing allowed this niece access to colonoscopies that she would otherwise not have known to access:

*“The number one benefit is it saves lives. And point in fact*,* had I not reached out and been persistent with my brother-in-law*,* and had he not had the conversation with his daughter*,* and she was diagnosed Lynch Syndrome positive*,* there would have been no reason in the world for her to go for a colonoscopy at 24-years-old until she had some overbearing symptoms that were probably too late to correct.”* – Participant 203, non-Hispanic/Spanish/Latino, male, Lynch syndrome positive, first tested in family.

### Theme 3. Reflections on strengths and limitations

Participants reflected on strengths and limitations of cascade testing based on their experiences and shared their views on how to improve the process of cascade testing. Areas suggested for improvement included increasing public awareness and education around Lynch syndrome and cascade testing and encouraging healthcare providers to give more detailed guidance about how to discuss cascade testing with family.

Participants described differing experiences with their clinicians. Some participants perceived their clinicians to have a greater, or lesser, understanding of Lynch syndrome and cascade testing. This perceived level of clinician-expertise played a role in determining the degree to which the participant needed to self-advocate and pursue their own research about Lynch syndrome and cascade testing.

*“The biggest thing for me was that the initial geneticist was so ‘pooh-phooey’ [sic.] that it just was - I had to do my own digging. I had to do my own research. I had to do my own figuring it out. I had to map my own way with the cascade testing because she was absolutely no help. I mean*,* it was a very scripted type situation*,* and that cascade testing was not a part of her script.”* – Participant 644, non-Hispanic/Spanish/Latino, female, Lynch syndrome positive, first tested in family.

Participants specifically described positive experiences with genetic counselors, who promoted cascade testing, provided education, and guided them in building a specific plan to approach family members.

*“I think that having a genetic counselor who was willing to sit with me and help me kind of go through it in my mind*,* it really helped me learn how to also share that information with my siblings. I think it’s important to whoever is initiating the conversation with their family*,* I think they should definitely have the tools to feel comfortable and knowledgeable of what they’re saying and how they’re saying it because I think in how you present something*,* it really makes a difference.”* – Participant 621, non-Hispanic/Spanish/Latino, female, Lynch syndrome positive, cascade tested.

## Discussion

Our results suggest that although experiences with cascade testing for Lynch syndrome are heterogenous, many participants expressed a desire for additional, targeted support during the process. Consistent with previous research, participants commonly expressed a belief that cascade testing had the power to save lives yet felt underprepared following their interactions with healthcare providers to communicate information to their at-risk relatives (Afaya et al. 2024; Srinivasan, Won et al. 2020b). Nuanced interventions are needed to ensure individuals are enabled and empowered to make informed decisions about predictive genetic testing.

Suggestions from participants included offering materials such as educational pamphlets, family letters, and verified online resources. These ideas have been echoed in previous research that suggests probands and family members favor of the use of written materials to facilitate cascade testing (Griffin et al. 2020; Heuvel et al. 2019). Previous research has also suggested having a number of diverse and accessible interventions may support increased uptake of genetic testing (Barrow et al. 2015; Bowen et al. 2021; Cragun et al. 2020; Dilzell et al. 2014; Petersen et al. 2018; Sermijn et al. 2016). Of note, previous findings suggest that individuals who receive educational materials during cascade testing are more likely to engage more with genetics services, and undergo predictive testing (Petersen et al. 2018). Based on the experiences of our participants who described disclosing genetic risk information to relatives without speaking face-to-face, it is worth considering how we might better facilitate family communication that is conducted via telephone, in writing, or online. Several studies have demonstrated the acceptability and usability of online and mobile-based outreach interventions to facilitate disclosure of genetic risk information (Aeilts et al. 2021; Haas et al. 2021; Pande et al. 2021; Pollard et al. 2020). We suggest there may be value in offering written support materials in conjunction with online support systems to best address the varied needs of families.

Participants felt strongly that cascade testing was important, and recognized uptake of predictive genetic testing has the potential to save lives. Our focus on the lived experiences of those who engaged with genetics services has been influenced by the prevailing moral framework surrounding genomics. Genetic testing is often framed within families as a moral imperative and duty to one’s family, and within the medical community as a responsible choice, which can lead to marginalization of those who declined to engage with cascade testing (Cowley 2016). This bias is reflected in our results, as participants often described relatives who exercised their right ‘not to know’ with some degree of negative judgement.

In a 2020 study investigating relatives’ readiness to undergo cascade genetic testing for hereditary cancer, 79.4% of living, untested first-degree relatives were in the precontemplation stage of change, meaning they were not yet ready to undergo genetic testing, despite awareness of the family variant (Bednar et al. 2020). Another study found that a communication aid did not improve uptake of hereditary cancer genetic testing among relatives, despite a high proportion of disclosure (Garcia et al. 2020). There is a disconnect between disclosure of information, subsequent engagement with genetics services, and uptake of genetic testing. The current approach results in a heterogeneity of experiences, potentially leading to suboptimal public health outcomes and perpetuating inequities in access to healthcare.

Bioethical arguments suggests a shift towards professional responsibility, suggesting that healthcare systems and providers have a moral responsibility to proactively and directly alert at-risk relatives to prevent harm, rather than the proband assuming the sole burden of disclosure (Grill and Rosén 2021). Recently, the European Society of Human Genetics has similarly endorsed a more supportive and proactive approach to cascade testing including optimising proband support, adopting greater directiveness, improving awareness of the nuance in medical confidentiality, and considering direct or digitally-mediated contact approaches (De Wert et al. 2026). There has been some encouraging evidence to imply the uptake of genetic testing increases when healthcare professionals assume responsibility for disclosure to relatives, however these findings are not universally consistent (Menko et al. 2018; Roberts et al. 2018; Srinivasan et al. 2020a, b; Suthers et al. 2011). Recent research suggests that the efficacy of direct contact methods may be more nuanced than this clear relationship (Menko et al. 2024). A combined approach in which family-led disclosure is complemented by healthcare-led disclosure, while remaining responsive to the family’s unique preferences, values, and needs, might pose a more effective, acceptable, and viable solution (Battistuzzi 2024; Grill et al. 2024; Mendes and Newson 2024; Tiller et al. 2025). Considering workforce constraints and scalability, appraisal suggests increasing feasibility of this approach in healthcare systems that have invested in digital communication tools (Bonini et al. 2024).

Crucially, our partcipants’ positive experiences with genetic counselors highlight that support needs to extend beyond informational resources. Automated tools such as chatbots have been shown to be broadly acceptable to patients and characterize a scalable, sustainable tool capable of supporting disclosure of risk information, promoting engagement with genetics services and therefore opportunity for informed decision making (Jones et al. 2021; Kaphingst et al. 2024; Nazareth et al. 2021; Riddle et al. 2021; Schmidlen et al. 2019; Siglen et al. 2022; Walters et al. 2023). However, in isolation, chatbots may be insufficient for addressing the complex and diverse needs of families, and should instead be considered for integration into a broader service provision model including optional telemedicine to maintain the relational benefits characteristic of genetic counseling.

### Strengths and limitations

This study has enabled comprehensive examination of rich data highlighting the lived experiences of individuals with or at-risk of Lynch syndrome. Lived experience of cascade testing in the context of Lynch syndrome has limited exploration in the literature, and our findings are novel in that they provide in-depth insight into the experiences and perspectives of this specific disease group. It should be noted that similar perspectives have been echoed in research focusing on patients with other inherited cancer predispositions, suggesting there are some universal themes around the current approaches taken to cascade testing for inherited cancer predispositions such as lack of support, which likely reflect constraints of current healthcare systems.

There are some limitations to our study. The perspectives of individuals who choose not to undergo cascade testing or do not have strong views one way or another were not included, representing a missed opportunity for important insight. Future work should also seek to understand the experiences of those who engage with genetic counselling and decide not to proceed with testing.

Through purposive sampling, the researchers from the larger study consented an ethnically diverse group representative of the wider US population, including the experiences and perspectives of non-White individuals we have historically failed to prioritize. Instead of generalizing from the perspectives of other populations, this study provides insight into lived experience by including Lynch-affected families from a variety of backgrounds. Participants were based in the USA, and responses reflected experiences with the local healthcare system. Adressing systemic inequities in healthcare requires prioritizing such critical investigations. Future research should also investigate the experiences of those outside of the USA to guide globally-appropriate implementation strategies.

## Conclusion

Lynch syndrome is an underdiagnosed hereditary cancer syndrome with established testing and surveillance recommendations shown to reduce mortality and burden of disease. Supporting cascade testing among at-risk relatives of individuals found to carry a Lynch-related MMR gene variant is one of the most important avenues to identify those most at risk of Lynch syndrome. Our findings suggest that experiences with cascade testing are varied, and support to families must be enhanced and tailored to suit their individualized needs. This includes support for family members’ ‘right not to know’, as availability of cascade testing can create a perceived moral imperitive to undergo genetic testing. Alternative models of service provision demonstrate the potential to provide tailored, accessible guidance for individuals and their families.

## Data Availability

No datasets were generated or analysed during the current study.
